# Clinical and Metabolic Characteristics and Follow-Up Results of Adrenal Incidentalomas: A 10-Year Experience

**DOI:** 10.7759/cureus.72221

**Published:** 2024-10-23

**Authors:** Yasin Arac, Guzin F Yaylali, Senay Topsakal, Canay Onder

**Affiliations:** 1 Acute Medicine, Queen Elizabeth Hospital Birmingham, Birmingham, GBR; 2 Diabetes and Endocrinology, Pamukkale University, Denizli, TUR; 3 Internal Medicine, Birmingham Heartlands Hospital, Birmingham, GBR

**Keywords:** adrenal adenoma, adrenal gland incidentaloma, adrenal glands, adrenal incidentaloma, adrenal incidentaloma investigations, autonomous cortisol secretion, cushing's syndrome, metabolic diseases, primary hyperaldosteronism

## Abstract

Objective: In this retrospective study, we aimed to investigate the clinical, hormonal, and radiological characteristics of patients with adrenal incidentalomas (AIs), to assess the prevalence of metabolic syndrome components in patients with AIs, and to determine whether changes in tumor size or hormonal activity occur during long-term follow-up in patients with AIs.

Methods: We retrospectively analyzed data from 384 patients diagnosed with AI between 2010 and 2020. Data regarding radiological, hormonal, and metabolic investigations in diagnosis and also during follow-ups were collected.

Results: A total of 384 patients (248 female, 64%) enrolled in this study. Of them, 348 (90.6%) of the masses were classified as adenomas, 31 (8.07%) as non-adenomas, and four (1.04%) were non-specified. The mean adenoma diameter was approximately 2 cm. Adenomas were found to be mostly on the left side (48.9%). Overall, 13.81% (n = 53) of the tumors were functioning, and among functional adenomas, nine patients (2.34%) had autonomous cortisol secretion, seven subjects (1.82%) had primary hyperaldosteronism, four subjects (1.04%) had pheochromocytomas, and 39 subjects (10.1%) had possible autonomous cortisol secretion. In patients with bilateral incidentalomas, the mean diameter of adenomas was higher than unilateral incidentalomas with a diameter of 24.22 mm in comparison to 20.36 mm (p > 0.05). Similarly, in patients with bilateral incidentalomas, systolic and diastolic blood pressure was higher than in unilateral incidentalomas (135.83 ± 20.27, 130.02 ± 18.84 and 79.25 ± 11.56, 78.52 ± 10.11, respectively, p > 0.05). Moreover, our study revealed that the frequency of diabetes, hypertension, and hyperlipidemia in patients with possible autonomous cortisol secretion was 37%, 63%, and 22.2%, respectively. Of 384 patients, 9.11% (n = 35) underwent surgery. The most common pathological finding was adrenocortical adenoma (n = 19, 54%). The median follow-up duration of patients was 48.91 months. Of 384 patients, 44.7% (n = 172) were followed up regularly with CT/MRI. During the follow-ups, the diameter of adenomas (11.6%) increased by more than 10 mm. Of 384 subjects, 56.7% (n = 218) of patients were followed with hormonal evaluation, six patients had developed possible autonomous cortisol secretion, and three patients had developed autonomous cortisol secretion. Moreover, one subject developed primary hyperaldosteronism.

Conclusions: In this retrospective study, we examined the clinical, radiological, and hormonal profiles of patients with AIs, finding results consistent with existing literature. The conversion to subclinical or clinical hypercortisolism was low, and no new cases of pheochromocytoma were observed, with only one case of primary hyperaldosteronism. Most of the adenomas showed minor growth in size over time, and a small percentage developed hormonal hyperfunctionality. Our study also identified an increased risk of diabetes, hypertension, hyperlipidemia, and metabolic syndrome in AI patients. Additionally, patients with unilateral adenomas had higher triglyceride levels and insulin resistance than those with bilateral adenomas.

## Introduction

Adrenal incidentalomas (AIs) are masses detected in the adrenal gland in imaging studies performed for any reason [1]. Previous studies have reported a prevalence of AIs ranging from 1% to 10%, which increases with age [2,3]. Clinicians should carefully evaluate patients with AIs by taking a detailed medical history and performing physical examinations and hormonal and biochemical tests to determine their hormonal functionality or potential risk of malignancy and metastasis [1,2]. The majority of AIs are benign non-functioning adrenal incidentalomas (NFAIs) [1,2], which mostly do not require surgery at the time of presentation [1]. However, currently, there is some debate regarding the optimal frequency and length of follow-up and which tests should be performed during follow-up. Recent European guidelines advise against routine repeat imaging and hormonal reassessment unless new symptoms emerge given that most AIs remain stable in size over time and only infrequently progress to hormone-secreting tumors [1,2].

A further issue complicating the treatment of patients with AI is the co-occurrence of metabolic conditions like diabetes mellitus (DM), hypertension (HT), hyperlipidemia (HL), and metabolic syndrome (MS) [3-5]. Some research studies investigating the co-occurrence of MS and AI have found that MS components are more common in NFAI patients compared to healthy controls [4,5]. In addition, several retrospective studies have suggested an increased prevalence of cardiovascular and metabolic risk factors associated with possible autonomous cortisol secretion (pACS), including HT, increased left ventricular mass, impaired glucose tolerance, and increased visceral fat, in patients with AI [3,6].

The objectives of this study are to investigate the clinical, hormonal, and radiological characteristics of patients with AI, to assess the prevalence of MS components in patients with AI, and to determine whether changes in tumor size or hormonal activity occur during long-term follow-up in patients with AIs. In these patients, we evaluated the comorbidities of patients with AIs across different age groups. We also compared the clinical and biochemical characteristics of unilateral and bilateral AIs.

## Materials and methods

This study was carried out at the Division of Endocrinology, Pamukkale University, Denizli, Turkiye. We retrospectively analyzed data from 384 patients diagnosed with AI between 2010 and 2020. Approval for this study was obtained from the Pamukkale University ethics committee (60116787-020/8337). While studying at Pamukkale University, Dr. Arac conducted research in collaboration with Dr. Yaylali and Dr. Topsakal. Dr. Onder later joined the team, assisting with data interpretation and editing of the article. This study included patients referred to our clinic with the diagnosis of AI, and only the patients with complete hormonal tests were included in the study. Patients below the age of 18 years and patients with a known history of malignancy were excluded. We recorded all patients’ age, gender, and comorbidities. All data were collected from patient records.

Information on DM was gathered if the patient had a prior diagnosis or had been on diabetes medications. In patients without known DM, the diagnosis was established according to the diagnostic criteria of the American Diabetes Association. Specifically, patients with at least one of the following were deemed to have DM: fasting plasma glucose ≥126 mg/dL, two-hour plasma glucose ≥200 mg/dL during oral glucose tolerance test (OGTT), glycosylated hemoglobin (HbA1C) ≥6.5%, or a random plasma glucose ≥200 mg/dL in a patient with classic symptoms of hyperglycemia or hyperglycemic crisis [7]. We gathered information on HT if the subject had a prior diagnosis of HT or was currently on anti-hypertensive medications. In patients without known HT, the diagnosis was established according to the European Society of Cardiology guidelines. HT is diagnosed if the systolic blood pressure is >140 mmHg and the diastolic blood pressure is >90 mmHg [8]. Information on HL was gathered for patients with a prior diagnosis or using anti-hyperlipidemic medications. In patients without a previous diagnosis of HL, the diagnosis was established according to the European Society of Cardiology guidelines; specifically, patients were deemed to have HL if their total cholesterol was >200 mg/dL and their low-density lipoprotein (LDL) cholesterol was >130 mg/dl or if isolated hypertriglyceridemia was present (triglycerides > 200 mg/dL) [9]. Patients were determined to have MS if they had three or more of the following: waist circumference >102 cm in men and >88 cm in women; serum triglyceride level ≥150 mg/dL; high-density lipoprotein (HDL) cholesterol <40 mg/dL in men and <50 mg/dL in women; blood pressure ≥130/85 mmHg; or fasting glucose ≥110 mg/dL [9]. Osteoporosis information was gathered for patients with their prior dual-energy X-ray absorptiometry (DXA) measurement (lowest T-score values below −2.5 SD was considered osteoporosis), according to World Health Organization (WHO) guidelines, or based on their use of osteoporosis therapy [10]. We measured the body mass index (BMI) and blood pressure and evaluated fasting plasma glucose, serum lipids, glycated hemoglobin A1c, and creatinine. Finally, the homeostatic model assessment for insulin resistance (HOMA-IR) was calculated as follows: fasting insulin (μU/ml) × fasting glucose (mmol/l)/22.5.

Patients underwent radiological, hormonal, and metabolic investigations in diagnosis and also during the follow-ups. The initial radiological examinations conducted included non-contrast computed tomography (CT), magnetic resonance imaging (MRI), and positron emission tomography (PET) CT. We recorded the location and diameter of all the patients’ adenomas. We defined "multiple adrenal lesions" as the presence of more than one identifiable lesion on either the left or right adrenal gland in patients, while explicitly excluding cases involving bilateral adrenal lesions. Patients with bilateral lesions were categorized separately and were not included in the "multiple adrenal lesions" group. The hormonal evaluations involved measuring cortisol levels after an overnight 1 mg dexamethasone suppression test (DST), urinary normetanephrine (normal range: 0-390 mg/day), urinary metanephrine (normal range: 0-261 mg/day), plasma renin activity (ng/ml/hour), and serum aldosterone levels (pg/ml). Patients whose cortisol levels were suppressed (≤1.8 μg/dL) following the 1 mg DST and with normal 24-hour metanephrine-normetanephrine levels in the urine and a plasma aldosterone/renin activity ratio <20 were considered to have a “non-functional adenoma.” Hypercortisolism was excluded if patients had serum cortisol levels lower than 1.8 g/dL after the 1 mg DST. Conversely, patients with values between 1.9 g/dL and 5 g/dL were categorized as having pACS, and those with values above 5 g/dL were considered to have autonomous cortisol secretion (ACS) [1]. In this study, pheochromocytoma was defined based on patients having three-fold elevated levels of urinary normetanephrine or metanephrine. We calculated plasma aldosterone/renin activity in patients with HT or hypokalemia, and if this value exceeded 20, we performed a saline infusion test. Post-infusion plasma aldosterone > 162 pmol/L confirmed the diagnosis of primary hyperaldosteronism (PHA). Following these investigations, if a patient’s adenoma was found to produce two different hormones, it was defined as a “bi-functioning adenoma,” whereas if it only produced one hormone, it was defined as a “functioning adenoma.”

For patients who were recommended to undergo follow-up, radiological evaluation with CT or MRI was performed six to 12 months after the initial diagnosis, and the patients then attended follow-ups yearly. Changes in the diameter of the adenoma were classified into four groups: an increase in diameter greater than 10 mm from baseline or the formation of a new adenoma; a change in diameter less than 10 mm or no change at all; a decrease in diameter; and the disappearance of the adenoma. We performed hormonal evaluations (i.e., 1 mg DST, 24-hour metanephrine-normetanephrine levels in urine, and the plasma aldosterone/renin ratio) in addition to the radiological evaluation.

We used SPSS version 15.0 (SPSS Inc., Chicago, IL) for statistical analysis. In our analysis, missing values were handled as listwise deletion. We conducted normality tests, specifically the Shapiro-Wilk/Kolmogorov-Smirnov test, to assess whether the data were normally distributed. The results indicated that the data followed a normal distribution for variables such as age, BMI, systolic and diastolic blood pressure, and biochemical characteristics (e.g., LDL, HDL cholesterol, triglycerides, and HOMA-IR). Therefore, we used mean ± standard deviation to summarize these data. The age of the patients was categorized into 18-39, 40-69, and ≥70 years. A comparative analysis was conducted using the chi-square test for categorical variables. For continuous data, Student's t-test and one-way ANOVA were applied to parametric data, while the Mann-Whitney U test was used for non-parametric data. A p-value < 0.05 was considered statistically significant.

## Results

In total, 384 patients, of whom 248 were female (64%), participated in this study. The majority of the patients were between the ages of 40 and 69 years (50.5%, 194), and the number of patients over the age of 70 was 174 (44.7%). The most common radiological imaging modality used at admission was CT (269, 71.9%). The baseline demographic characteristics of the patients and the location of the adrenal adenomas are presented in Table 1.

**Table 1:** Baseline demographical characteristics and initial imaging characteristics of the patients.

	All subjects		18–39 years		40–69 years		≥70 years	
	n	%	n	%	n	%	n	%
All patients	384	100%	16	100%	194	100%	174	100%
Female	248	64.5%	13	81.2%	137	70.6%	97	55.7%
Male	136	35.5%	3	18.8%	57	29.4%	77	44.3%
Right-sided	128	33.3%	7	43.7%	69	35.5%	58	33.3%
Left-sided	188	48.9%	7	43.7%	95	48.9%	82	47.1%
Bilateral	68	17.7%	2	12.5%	30	15.4%	34	19.5%

Radiological evaluation of the adrenal tumors

During the initial radiological evaluations, we classified 348 (90.6%) of the masses as adenomas, 31 (8.07%) as non-adenomas, and four (1.04%) as non-specified. The mean adenoma diameter was 21.58 ± 12.06 mm with the range of 5-71 mm. Adenomas were predominantly on the left (n = 188, 48.9%), 17.7% (n = 68) of the AIs were bilateral, and 33.3% (n = 128) of them were on the right. Additionally, 16 patients (4.16%) had multiple adrenal lesions.

Hormonal assessment of the adrenal tumors

Overall, 13.81% of the tumors (n = 55) were functioning. There were 39 adenomas (10.1%) with pACS, seven adenomas (1.82%) with hyperaldosteronism, and four adenomas (1.04%) with pheochromocytomas. Of these, nine adenomas (2.34%) had ACS. Finally, the number of bifunctional adenomas was four (1.04%). Information regarding the initial imaging and hormonal results for the patients is presented in Table 2.

**Table 2:** Information from radiological and hormonal assessments of patients with incidentally discovered adrenal tumors.

	All subjects		18–39 years		40–69 years		≥70 years	
	n	%	n	%	n	%	n	%
All patients	384	100%	16	100%	194	100%	174	100%
Right-sided	128	33.3%	7	43.7%	69	35.5%	58	33.3%
Left-sided	188	48.9%	7	43.7%	95	48.9%	82	47.1%
Bilateral	68	17.7%	2	12.5%	30	15.4%	34	19.5%
Non-multiple	370	96.3%	16	100%	189	97.4%	167	95.9%
Multiple	14	3.6%	0	0%	5	2.5%	7	4%
Multiple right	5	1.3%	0	0%	2	1%	1	0.5%
Multiple left	9	2.3%	0	0%	3	1.5%	6	3.4%
Adenoma	348	90.6%	11	68.7%	181	93.2%	157	90.2%
Non-adenoma	31	8%	5	31.2%	12	6.1%	13	7.4%
Non-specified	4	1%	0	0%	1	0.5%	4	2.2%
Non-functioning adenoma (unilateral)	264	68.7%	7	43.7%	189	97.4%	68	39%
Non-functioning adenoma (bilateral)	67	17.4%	1	6.2%	49	25.2%	17	9.7%
Bi-functioning	4	1%	0	0%	3	1.5%	1	0.5%
Pheochromocytoma	4	1%	1	6.2%	3	1.5%	0	0%
Possible autonomous cortisol secretion	39	10.1%	2	12.5%	15	7.7%	22	12.6%
Autonomous cortisol secretion	9	26.4%	1	6.2%	5	2.5%	3	1.7%
Aldosteronoma	7	1.8%	0	0%	4	2%	3	1.7%

Comorbidities of patients with AIs

In our study, DM was identified in 128 (33%) of the patients, HT in 182 (47.4%), MS in 95 (24.70%), HL in 108 (28.10%), cardiovascular disease in 49 (12.80%), and osteoporosis in 43 (11.02%). The comorbidities of the patients across the age groups are shown in Table 3. On the other hand, the prevalence of comorbidities in patients with primary aldosteronism (pACS) was found to be as follows: DM was present in 37% of patients, HT in 63%, and HL in 22.2%.

**Table 3:** Comorbidities of patients with adrenal incidentalomas across the age groups.

	All subjects		18–39 years		40–69 years		≥70 years	
	n	%	n	%	n	%	n	%
Metabolic syndrome	95	24.7%	2	12.5%	63	32.4%	30	17.2%
Diabetes mellitus	128	33.0%	2	12.5%	91	46.9%	35	20.1%
Hypertension	182	47.4%	5	31.2%	117	60.3%	60	34.4%
Hyperlipidemia	108	28.1%	1	6.2%	72	37.1%	35	20.1%
Cardiovascular disease	49	12.8%	0	0%	25	12.8%	24	13.7%
Osteoporosis	43	11.02%	0	0%	27	13.9%	16	9.1%

Follow-up results

In our study, the median follow-up duration of patients was 48.91 months. While certain patients had complete hormonal follow-up data, others only had complete radiological follow-up data. A total of 166 patients' hormonal data and 212 patients' size data were lost to follow-up.

Changes in Adrenal Adenoma Size and Malignant Transformation

Overall, 172 patients (44.7%) had complete radiological follow-up data. In 20 of the patients (11.6%), the diameter of the adenomas increased by more than 10 mm during the follow-up period. Of these 20 patients, only two required surgery due to the increasing size of the adenomas and intermediate radiological features. The pathology results for these patients were adrenocortical adenoma and an adrenal cyst. Conversely, in 15 patients (8.7%), the diameter of the adenomas has decreased. During the follow-up period, no patients with a benign tumor developed adrenocortical carcinoma.

Changes in Hormone Production

The follow-up data on changes in adenoma functionality were complete in 218 of the patients (56.7%). Of these patients, six developed pACS, and three developed ACS. Due to the presence of comorbidities, one patient in each of these two groups underwent surgery, and the pathology reports from both of these cases revealed adrenocortical adenoma. No patients had elevated 24-hour fractionated urine metanephrine or normetanephrine concentrations after five years of follow-up. Furthermore, one subject developed PHA. Tables 4, 5 display the information about the changes in hormone production and adenoma diameter.

**Table 4:** Follow-up evaluation of adrenal adenomas' diameter across the age groups.

	All subjects		18-39 years		40-69 years		≥70 years	
Follow-up duration (months)	48.91 ± 34.11		49.56 ± 45.74		47.21 ± 30.05		50.73 ± 37.20	
	n	%	n	%	n	%	n	%
Increase ≥10 mm in diameter	20	11.6%	3	18.7%	11	5.6%	7	4%
Increase <10 mm in diameter	137	79.6%	2	12.5%	67	34.5%	68	39%
Decrease in diameter	15	8.7%	1	6.2%	7	3.6%	7	4%

**Table 5:** Hormonal follow-up evaluation of adrenal adenomas across the age groups.

	All subjects		18-39 years		40-69 years		≥70 years	
Follow-up duration (months)	48.91 ± 34.11		49.56 ± 45.74		47.21 ± 30.05		50.73 ± 37.20	
	n	%	n	%	n	%	n	%
Development of Cushing’s syndrome	3	1.3%	0	0%	2	1%	0	0%
Development of autonomous cortisol secretion	6	2.7%	0	0%	3	1.5%	3	1.7%
Development of pheochromocytoma	0	0%	0	0%	0		0	0%
Development of primary hyperaldosteronism	1	0.45%	0	0%	1	0.5%	0	0%

Comparison of bilateral and unilateral adenomas

Table 6 demonstrates the clinical and biochemical characteristics of the patients in this study and gives a comparison of unilateral and bilateral adenomas. Overall, 67 (12.7%) of the non-functional adenomas were bilateral, and 264 (68.4%) of the adenomas were unilateral. The mean adenoma diameter was greater in patients with bilateral AIs compared to those with unilateral AIs (i.e., 24.22 mm vs. 20.36 mm, respectively; p > 0.05).

**Table 6:** Clinical and biochemical characteristics of unilateral and bilateral adrenal incidentalomas. HDL: high-density lipoprotein; LDL: low-density lipoprotein; HOMA-IR: homeostatic model assessment for insulin resistance.

	Unilateral (n = 264)	Bilateral (n = 67)	P-value
Age (years)	61.73 ± 11.93	62.80 ± 12.19	>0.05
BMI (kg/m^2^)	30.72 ± 5.41	28.52 ± 5.06	>0.05
Systolic blood pressure (mmHg)	130.02 ± 18.84	135.83 ± 20.27	>0.05
Diastolic blood pressure (mmHg)	78.52 ± 10.11	79.25 ± 11.56	>0.05
Glucose (mg/dL)	117.56 ± 43.67	109.67 ± 21.49	<0.05
Creatinine (mg/dL)	0.76 ± 0.19	0.77 ± 0.19	>0.05
HDL (mg/dL)	51.73 ± 16.32	53.45 ± 17.56	<0.05
LDL (mg/dL)	119.18 ± 36.88	121.26 ± 37.46	>0.05
Total cholesterol (mg/dL)	201.15 ± 43.07	200.86 ± 40.42	>0.05
Triglycerides (mg/dL)	151.62 ± 84.60	128.38 ± 55.69	<0.05
HOMA-IR	3.86 ± 2.76	2.65 ± 1.40	<0.05
Hemoglobin A_1c_ (%)	6.54 ± 1.44	6.22 ± 0.89	>0.05

Patients with bilateral AIs showed higher (p > 0.05) systolic and diastolic blood pressure compared to those with unilateral AIs. Specifically, the systolic blood pressures of these groups were 135.83 ± 20.27 mmHg and 130.02 ± 18.84 mmHg, respectively, and the diastolic blood pressures were 79.25 ± 11.56 mmHg and 78.52 ± 10.11 mmHg, respectively. Conversely, no differences were detected between the unilateral and bilateral groups in terms of DM, HT, HL, cardiovascular disease, and MS.

Glucose levels were found to be significantly higher (p < 0.05) in patients with unilateral adenoma compared to patients with bilateral adenomas (i.e., 117.56 ± 43.67 versus 109.67 ± 21.49). HDL levels were significantly higher (p < 0.05) in patients with bilateral adenomas in comparison with unilateral adenomas (i.e., 53.45 ± 17.56 versus 51.73 ± 16.32).

Comparison of functioning and non-functioning adenomas

Functioning AIs had a larger baseline diameter than NFAIs (i.e., 29.37 ± 12.33 versus 21.58 ± 12.06, p > 0.05). Patients with pACS had a mean systolic blood pressure of 138.07 ± 20.69, which was higher (p > 0.05) than that of patients with non-functioning AIs. Moreover, our results showed that the prevalence of DM, HT, and HL in patients with pACS was 37%, 63%, and 22.2%, respectively.

Histological findings of patients who underwent surgery

Of the 384 patients in this study, 9.11% (n = 35) underwent surgery. The criteria for surgery were as follows: mass size, malignant criteria, and hormone secretion.

Table 7 displays the histological findings for the AIs of the patients in this work who underwent surgery. The most common pathological finding was adrenocortical adenoma, reported in 19 of the cases (54%). Additionally, five patients (14.0%) were found to have pheochromocytoma, and one was found to have an adrenal metastasis.

**Table 7:** Histological data of the patients who underwent surgery.

	All subjects		18–39 years		40–69 years		≥70 years	
	n	%	n	%	n	%	n	%
Pheochromocytoma	5	1.3%	3	18.7%	2	1%	0	0%
Adrenal cysts	1	0.2%	1	6.2%	0	0%	0	0%
Myelolipomatous changes	3	0.7%	0	0%	3	1.5%	0	0%
Adrenal metastasis	1	0.2%	0	0%	0	0%	1	0.5%
Adrenocortical carcinoma	1	0.2%	0	0%	1	0.5%	0	0%
Corticomedullary mixed tumor	1	0.2%	1	6.2%	0	0%	0	0%
Adrenocortical adenoma	19	4.9%	2	12.5%	12	6.1%	5	2.8%
Nodular/plexiform schwannoma	1	0.2%	0	0%	1	0.5%	0	0%
Pleomorphic leiomyosarcoma	1	0.2%	0	0%	1	0.5%	0	0%
Nodular hyperplasia	1	0.2%	0	0%	0	0%	1	0.5%
Caseous granuloma	1	0.2%	0	0%	1	0.5%	0	0%

## Discussion

In this study, the findings indicate that DM, HT, and MS are more common in AI patients than in the general population based on the population prevalence data. Even though previous studies showed that the development of PHA is very unusual in patients with AI, our study revealed the development of PHA in one patient.

Autopsy and radiology-based studies primarily detect AIs in women between the ages of 50 and 70 years old [11]. In our study, 50.5% (n = 194) of the cases were in patients aged 40-69 years, and 44.7% (n = 174) of the patients were aged 70 years and over. We found that 64.5% (n = 248) of the patients we investigated with AIs were women, in line with the findings of previous studies showing a higher prevalence of AIs among women than men [12,13]. In this study, the most commonly performed radiological examination at the time of admission was CT, which was conducted in 71.9% (n = 269) of cases, followed by MRI, which was conducted in 25% (n = 96) of cases. Similarly, previous studies have reported that CT is the most frequently used imaging modality for such patients [13,14].

One of the key issues regarding AI is detecting the risk of malignancy, for which size and radiological findings are important. In a study conducted by Kolanska et al. (2010), the mean tumor size was found to be 24.26 ± 9.02 mm [15], and Kim et al. (2014) reported a mean size of 27.3 ± 18.9 mm [16]. In line with this literature, the mean adrenal tumor size in our study was 21.58 ± 12.06 mm. Furthermore, the previous literature indicates that the risk of malignancy rises with increasing tumor size. For example, in a study conducted in Italy by Mantero et al. (n = 887), it was revealed that 90% of adrenocortical carcinomas had a diameter greater than 4 cm at presentation [12].

The literature presents varying data concerning the localization of AIs [17]. Our study revealed that 33.3% (n = 128) of the adenomas were on the right, and 48.9% (n = 188) were on the left. Furthermore, in our study, 82.2% (n = 316) of the adenomas were found to be unilateral, and 17.7% (n = 68) were bilateral. The frequency of bilateral adenomas was similar to that reported in the literature; indeed, in the studies of Olsen et al. and Barzon et al., the frequencies of bilateral adenomas were reported to be 19% and 15%, respectively [18,19]. Previous studies have also compared the sizes of unilateral and bilateral adenomas, but different results have been obtained. For instance, in a study conducted in Turkiye, bilateral adrenal adenomas were found to be larger than unilateral adenomas [14]. In our study, we compared unilateral and bilateral non-functional adenomas and did not find any significant difference in terms of their diameter (24.22 mm with bilateral AIs vs. 20.36 mm with unilateral AIs; p > 0.05). Moreover, our study revealed that 3.64% (n = 14) of the patients had multiple adenomas, whereas this prevalence was 16.8% in the study of Çömlekçi et al. [13].

Imaging characteristics alone are insufficient to reliably distinguish whether a mass is functional. Therefore, all patients diagnosed with an AI should be assessed for the AI’s functional activity using endocrine biochemical testing regardless of radiological suspicion [12]. In a study conducted by Kastelan et al. in 2015 with 318 patients with AI, 74.6% of the cases were reported as nonfunctional adenoma, 11.3% as subclinical Cushing’s syndrome, 5.3% as pheochromocytoma, and 3.7% as hyperaldosteronism [20]. Furthermore, another study in Japan reported that 50.8% of 3,672 cases of AIs were nonfunctional adenomas, 10.5% were subclinical Cushing’s syndrome, 8.5% were pheochromocytoma, and 5.1% were hyperaldosteronism [21]. In the study of Çömlekçi et al., 73.5% of the patients with AIs were found to have non-functional adenoma, 12% to have subclinical Cushing's syndrome, 5.9% to have pheochromocytoma, 4.4% to have Cushing's syndrome, and 4.4% to have PHA [13]. In our study, we classified 331 (81.2%) AIs of patients as non-functional and 53 as functional. Additionally, pACS was detected in 39 (10%) of the adrenal masses, Cushing's syndrome in nine (2.3%), pheochromocytoma in four (1.04%), and hyperaldosteronism in seven (1.82%).

The literature contains a limited number of studies comparing the biochemical data of bilateral and unilateral NFAIs. In our study, the unilateral group exhibited significantly higher triglyceride values (p < 0.05). Additionally, glucose and HOMA-IR levels were found to be significantly higher in the unilateral group (p < 0.05). However, these results were not clinically significant as they did not affect the diagnosis of HL or DM. These results differ from those of Patrova et al., who found increased DM frequency in the group with bilateral adenomas [22].

Literature differs in terms of comorbidities in patients with AIs. In the 2010 study of Kolanska et al. [15], the mean BMI of patients with AIs was 28.77 ± 4.71 kg/m2, and in our study, the mean BMI was found to be 30.30 ± 5.35 kg/m2. In our study, we identified 128 (33%) patients with DM, 182 (47.4%) with HT, 95 (24.70%) with MS, 108 (28.10%) with HL, and 49 (12.80%) with cardiovascular disease. In terms of the population prevalence of these diseases, the Turkish Ministry of Health conducted the Chronic Diseases Risk Factors Survey (CDRFS) in 2011 and reported that 24% of the population had HT, 12.5% had HL, 11% had DM, and 23% had MS [23]. Therefore, given these prevalence estimates, it seems that DM, HT, and MS are more common in AI patients than in the general population [5,24,25]. Previous studies have suggested that AI heightens the risk of cardiovascular risk factors such as insulin resistance, DM, HT, and dyslipidemia [3,6], but this effect remains controversial because the age of the patients at diagnosis may also influence these comorbidities; indeed, diagnosis of AI peaks in people aged 50 to 70 years old. However, a comparison between our study’s comorbidity prevalence data and the prevalence of such diseases in the wider population aged 50-70 years highlights the increased risk of comorbidities in patients with AI.

In the study of Kastelan et al., DM was reported in 27.9% of patients with AI and HT in 69.3% of these patients [20]. Additionally, in a study by Majnik et al., the prevalence of type 2 DM was higher in patients with bilateral lesions, whereas the prevalence of HT and dyslipidemia was similar between bilateral and unilateral groups [26]. Meanwhile, in the study by Vassilatou et al., the frequency of HT was higher in patients with bilateral versus unilateral AIs [27].

Despite some publications suggesting a higher frequency of ACS in patients with bilateral compared to unilateral adenomas, this claim remains controversial. In our study, it was found that ACS was most common in patients with unilateral adenomas. Additionally, it has previously been reported that the risk of ACS increases with greater adenoma diameter, and ACS is generally observed more frequently in individuals with adenomas with diameters greater than 3 cm [27].

In this study, our average follow-up time was 48 months (48.91 ± 34.11). Previous AI follow-up studies have reported varying outcomes in terms of changes in adenoma mass size and hormonal secretion. In the current study, over the follow-up period, 157 out of the 384 patients showed an increase in adenoma mass size, with 20 (11.6%) showing a diameter increase of greater than 10 mm and 137 (79.6%) showing an increase of less than 10 mm. In 15 (8.7%) patients, we detected a reduction in the size of the adenomas over the follow-up period. In a previous study of 1,149 patients who were followed for four years, it was reported that CT was not needed in the follow-up of non-functional masses less than 4 cm in diameter and that these masses were rarely functional [28].

During the follow-up in the current study, we evaluated our cases in terms of changes in hormonal production of the adenomas (n = 218). During the follow-up, the development of Cushing’s syndrome was identified in three patients (1.3%), pACS in six patients (2.7%), and PHA in one patient (0.45%), but no patients developed pheochromocytoma. Other studies have reported the development of hyperfunctionality in approximately 5% of cases [14]. Furthermore, prior studies evaluating hormonal changes during the follow-up period have mostly reported the development of ACS and pACS. In the literature, the development of PHA is very rare, whereas our study showed the development of PHA in one patient. Moreover, in our study, two patients with pACS showed progression to Cushing’s syndrome.

One limitation is the use of general population prevalence data to compare comorbidities in patients with AIs. Since the majority of our cohort is aged 50-70 years, this comparison may be skewed by the age-related prevalence of conditions such as DM, HT, and MS. Ideally, a matched control group of similar age demographics would provide a more accurate assessment. Another limitation of this study is the significant loss of the follow-up data. This loss may limit our ability to fully evaluate the relationship between AIs and associated follow-up changes, potentially impacting the study's conclusions.

One of the key strengths of this study is its design as a comprehensive observational analysis, allowing for the collection of detailed data across multiple aspects of patients with AIs. By examining clinical, radiological, and hormonal characteristics, as well as the prevalence of metabolic syndrome components and the comparison of unilateral versus bilateral AIs, this study provides a comprehensive view of AIs. This thorough approach contributes valuable insights to the existing literature on AIs and their associations with comorbidities.

## Conclusions

In this retrospective study, we investigated the clinical and radiological characteristics and hormonal status of patients with AIs. Most of the findings are similar to the previous literature. The main findings of our study are that the conversion rate of patients with AIs to subclinical or clinical hypercortisolism was low and that there were no new cases of pheochromocytoma. Only one subject developed primary hyperaldosteronism. The follow-up duration of our study revealed that while most patients experienced minor increases in tumor size, a small percentage developed hormonal hyperfunctionality. Additionally, our study revealed an increased risk of developing DM, HT, HL, and MS in patients with AIs. Finally, we found that patients with unilateral adenomas exhibited significantly higher triglyceride levels and insulin resistance compared to those with bilateral adenomas, suggesting distinct metabolic profiles that warrant further investigation.
